# Disentangling the Optoelectronic Behavior of Lead Iodide Governed by Two-Dimensional Electron Confinement

**DOI:** 10.1021/acsami.4c10507

**Published:** 2024-10-15

**Authors:** Hamida Gouadria, Fernando Aguilar-Galindo, Jesús Álvarez-Alonso, Juan José de Miguel, Sergio Díaz-Tendero, María José Capitán

**Affiliations:** † Departamento de Física de la Materia Condensada, Universidad Autónoma de Madrid, 28049 Madrid, Spain; ‡ Departamento de Química, Universidad Autónoma de Madrid, 28049 Madrid, Spain; § Institute for Advanced Research in Chemistry (IAdChem), Universidad Autónoma de Madrid, 28049 Madrid, Spain; ∥ Física de Sistemas Crecidos con Baja Dimensionalidad, UAM, Unidad Asociada al CSIC por el IEM, DP , 28006 Madrid, Spain; ⊥ Instituto de Ciencia de Materiales “Nicolás Cabrera”, Universidad Autónoma de Madrid, 28049 Madrid, Spain; # Condensed Matter Physics Center (IFIMAC), Universidad Autónoma de Madrid, 28049 Madrid, Spain; ∇ Instituto de Estructura de la Materia IEM-CSIC, c/Serrano 121, 28006 Madrid, Spain

**Keywords:** optoelectronics, electron confinement, stability, photocurrent generation, band bending, 2D-system, PbI_2_

## Abstract

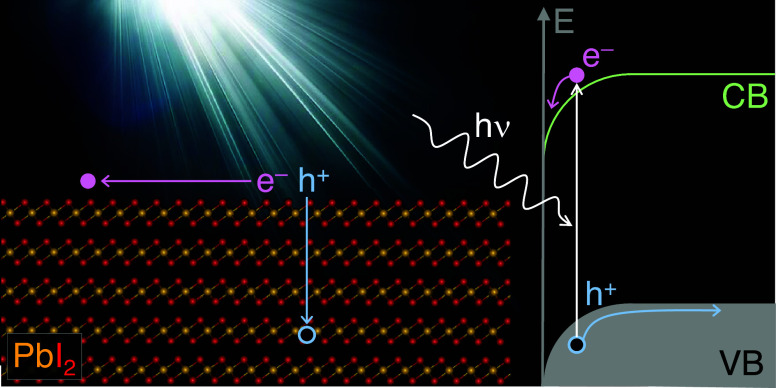

We present a joint experimental and theoretical study for complete spectroscopic characterization and optoelectronic properties of lead iodide. Experimentally, we combine X-ray diffraction experiments to elucidate the structure with photoelectron spectroscopy to explore its electronic structure. Computationally, simulations are performed in the frame of density functional theory. We show that PbI_2_ presents a two-dimensional layered structure and exhibits a large transient photocurrent effect under visible light illumination, which are compatible with the surface photovoltage scenario. The transient photocurrent has an extremely long lifetime: when the sample is lightened with visible light, it shows very long relaxation times and, consequently, huge charge carrier diffusion lengths. We explain this anomalous behavior with the slow carrier mobility of holes and electrons caused by the 2D electron confinement in the layered material. Our results can be used as a simple model for understanding the optoelectronic properties of more complex 2D hybrid perovskites.

## Introduction

In recent years, lead iodide (PbI_2_) has emerged as a promising material for room-temperature detection of X-ray, alpha, and γ radiations as well as for imaging and luminescence applications, among others.^1−4^ The interest in its optoelectronic properties was further boosted from the field of photovoltaics with the revolutionary appearance of metal halide-based hybrid organic–inorganic perovskite solar cells,^5^ for which PbI_2_ constitutes a precursor material.^6^

More recently, from a more fundamental point of view, a great amount of research is being dedicated to the study of two-dimensional (2D) materials^7^ due to the wealth of novel phenomena that are expected to be found in them.^8,9^ Among all of the different types of 2D materials, transition-metal dichalcogenides consist of slabs formed by single atomic layers of the transition metal sandwiched between single layers of chalcogen atoms. These trilayers are then stacked and loosely bounded by weak van der Waals interactions.^10^ The PbI_2_ structure can be also visualized as 2D slabs stacking, where each PbI_2_ slab consists of a Pb layer embedded between two I layers, and Pb is coordinated to six I atoms forming nearly an octahedron, while I is bonded to three Pb atoms in the trigonal pyramid geometry. Consecutive slabs are weakly bound through I–I van der Waals interactions. Thus, in recent years, PbI_2_ has gained new interest together with graphite as a 2D exfoliation material.^11,12^ Among the different exfoliable 2D materials, the family of 2D semiconductors is especially relevant for their applications. Many of the properties of this material are sensitive to dimensionality effects. For instance, low-frequency interlayer phonons have an influence on the electron–phonon interactions and the luminescence spectra.^4^ Thus, the family of 2D semiconductors is especially relevant for applications as their intrinsic band gap makes them suitable for different electronic and optoelectronic devices.^13^

Furthermore, an in-depth study of PbI_2_ is also key for understanding the optoelectronic properties of 2D hybrid organic lead-halide perovskites which currently play a highly prominent role for their technological applications.^6,14,15^ These 2D perovskites are characterized by high light capture-cross sections, remarkably long carrier lifetimes, and high collection efficiencies, yielding power conversion efficiency values as high as 22%.^16,17^ However, a fundamental issue that remains unresolved is the role of the charge carrier confinement within the PbI_2_ planes in the performance of these devices^18−21^ and their intrinsic electronic properties. This is precisely the main objective of the present work.

Despite its outstanding qualities, few works have investigated the key properties of PbI_2_ as a photoelectrode, including carrier diffusion length, band edge positions, and dynamics of interfacial recombination.^22,23^ The performance of PbI_2_-based light detectors cannot be fully understood unless the role of 2D confinement on its electronic and optical properties is determined. In this context, we have thoroughly studied the above-mentioned optoelectronic properties of PbI_2_, as well as its dependence on incident visible light, in a joint experimental-theoretical frame. We show that PbI_2_ presents an interesting photocurrent transient behavior, thus broadening its technological application fields beyond photodetection and opening new perspectives in, for example, photocatalysis and photovoltaic electronic materials.

## Results and Discussion

### Structure, Electronic Characterization, and Stability Under Vacuum

**Figure 1:**
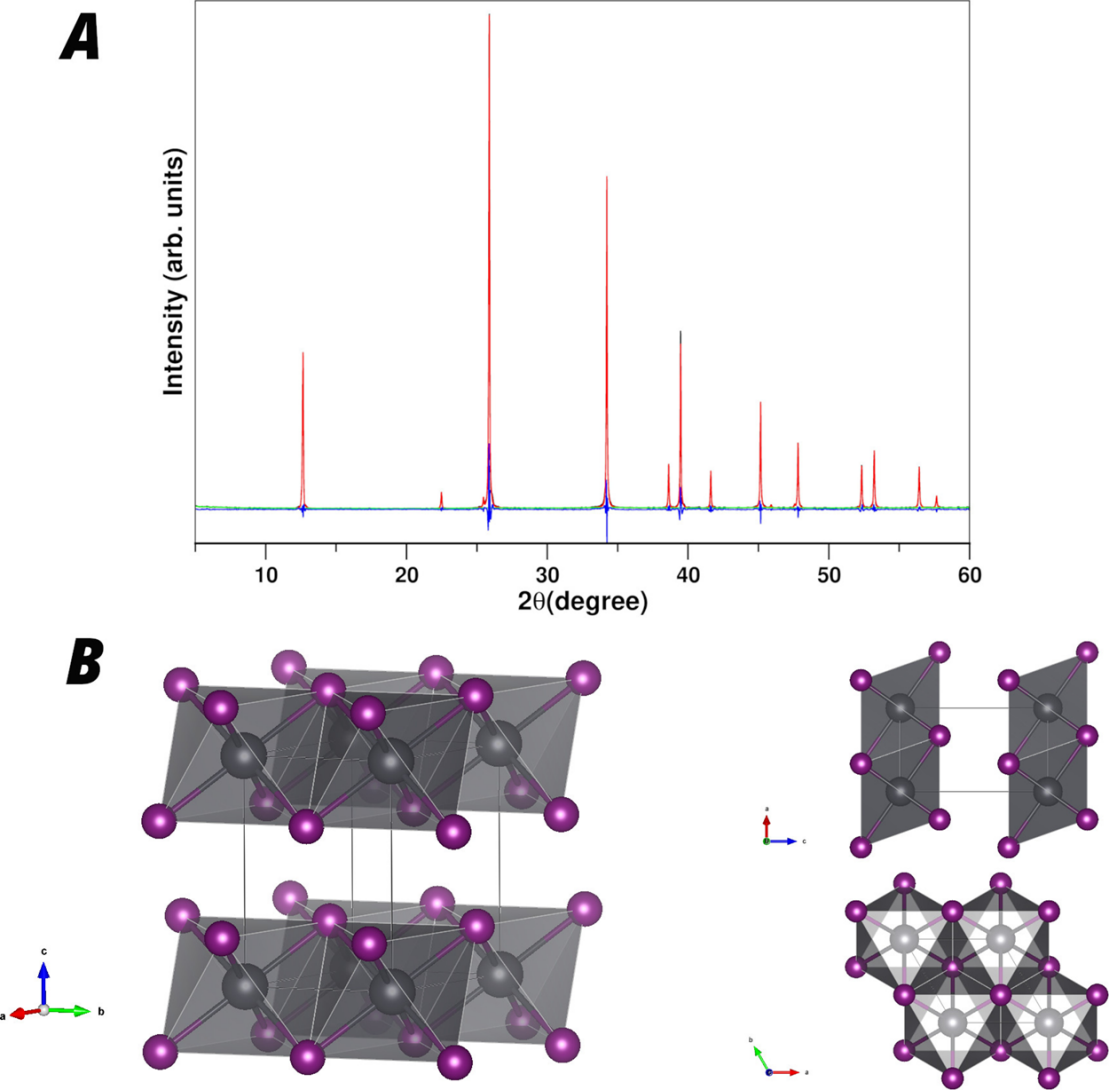
(A) X-ray diffraction diagram and its fit. (B) Sketch of the resulting fit structure.

In this section, we show the structural and electronic characteristics of the PbI_2_ sample and its stability under the experimental conditions. The measured X-ray diffraction patterns for the studied PbI_2_ powder samples are shown in Figure 1. The diffraction patterns were fitted for structure determination. The resulting fit structure is trigonal with *a* = *b* = 4.5605 Å and *c* = 6.9852 Å cell parameters with the *P*3̅*m*1 symmetry space group 164, in agreement with the literature.^24^Figure 1B shows a sketch of the atom arrangement resulting from the X-ray diffraction fit. The structure has an atomic arrangement formed by 2D PbI_2_ planes with the Pb placed at the center of I-octahedral sites and I-octahedral share faces. The structure shows an AB stacking order determining the exfoliation character of PbI_2_. The 2D inorganic plane is also present in many of 2D lead-iodide perovskites, making the determination of the 2D confinement influence on the electronic behavior of this 2D inorganic plane very interesting in order to clarify the electronic properties of these 2D-organic–inorganic perovskites.

The computed distances and angles agree with the experimentally measured distances (error below 1.5%), making us confident on the level of theory here employed. The calculated electronic structures and other electronic properties of the bulk and surface are shown and discussed in section 2.

**Figure 2:**
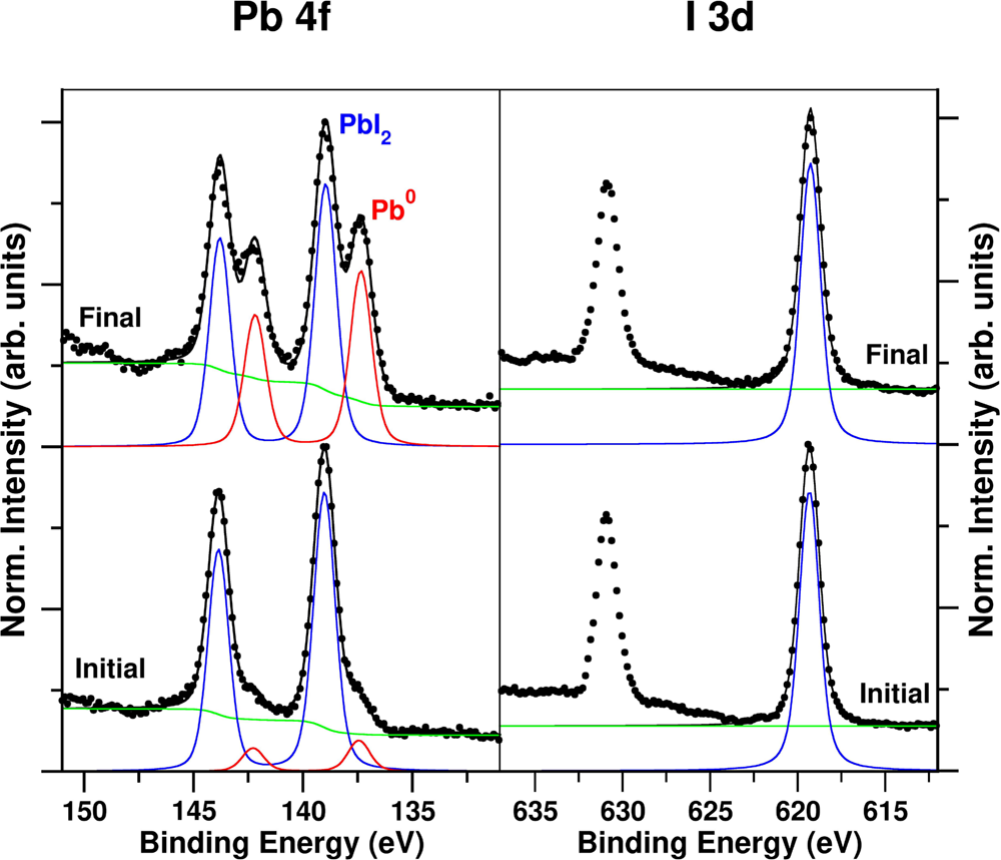
Pb 4f and I 3d XPS spectra region and their fit for the PbI_2_ fresh sample (lower spectra) and aged sample, 2 days after introduction into a UHV environment (top spectra).

The measured X-ray photoemission spectroscopy (XPS) spectra are listed in Figure 2. The total XPS spectra show, in addition to the Pb and I coming from the sample, the presence of Ag and residual traces of C, O, and N which are, all of these, associated with the Ag colloid used as a powder glue (not shown here). We have checked that the PbI_2_ powder is perfectly stable with time in the atmospheric environment (see Figure S1 in the Supporting Information). Neither structural nor electronic changes are observed in electronic spectroscopy with time when the sample is stored in the room environment. However, when it is set in a UHV chamber, the associated extreme conditions of the spectroscopy setup induces a sample aging that is reflected in changes in the electronic properties. The spectroscopy experimental setup requires vacuum conditions with pressure lower than 10^–10^ mbars, and in such conditions, the sample evolves with time. In the same direction, samples kept for several days under vacuum without X-ray illumination show also the same kind of sample degradation. On the other hand, the spectra of samples measured by X-ray diffraction after long time (above 2 days) do not show any changes. We observed the presence of iodine in the residual vacuum of the UHV chamber after the sample measurement. Thus, we can assume that the evolution of the sample is driven by the ability of iodine to sublimate under a UHV environment, and it is not driven by the experimental X-ray illumination. Thus, we distinguish between the fresh sample, which has been just introduced under vacuum, and the aged sample, which has been at least 1 day under a UHV environment.

In the fresh PbI_2_ powder sample, both Pb 4f and I 3d peaks are identified and can be well fitted with a Doniach-Sunjic combination of Lorentzian and Gaussian lineshapes peak (see Figure 2). The fresh sample shows a main component in both Pb 4f and I 3d spectra. Their peak positions are in good agreement with the literature for PbI_2_.^25,26^ The Pb 4f edge in the fresh PbI_2_ sample has a main peak split into two located at 139.0 and 143.8 eV (for Pb 4f_7/2_ and Pb 4f_5/2_, respectively) with a peak spin–orbit splitting of 4.8 eV. In the right panels of Figure 2, we show the region corresponding to I 3d, in particular the I 3d_5/2_ and I 3d_3/2_ peaks, located at 619.3 and 630.9 eV, respectively, with a spin–orbit splitting of 11.6 eV. These peak positions and splitting are consistent with the standard data in the literature.^25−27^ In the Pb 4f edge of the fresh sample, we can observe small features located at a lower BE. They are associated with low traces corresponding to the appearance of metallic lead (≃ 7% with respect to the PbI_4_ Pb 4f peak area). These secondary Pb peaks are related to the time spent introducing the sample into the UHV chamber (approximately 2 h) as we will undoubtedly demonstrate later with the aged sample.

The measured spectra after 2 days under vacuum are shown in the upper panels of Figure 2. As we have already pointed out, a sample under UHV conditions ages with time, and in the XPS spectra, several peaks appear in the Pb 4f region. In particular, the two Pb 4f peaks, Pb 4f_7/2_ and Pb 4f_5/2_, present two contributions each: one of them at the positions as those showed in the fresh sample, which are associated with PbI_2_, and the second one placed at 137.3 and 142.1 eV (for Pb 4f_7/2_ and Pb 4f_5/2_, respectively). The new contributions present the same spin–orbit peak splitting of 4.8 eV and are shifted 1.7 eV toward lower binding energy (BE) with respect to the PbI_2_ peaks. They are assigned to the presence of Pb^0^ atoms (Pb-metal) in the aged sample.^25^ In the fresh sample, we already identified a small contribution of this Pb-metal XPS peak corresponding to traces of metallic Pb atoms. Thus, the PbI_2_ powder is not stable under experimental UHV conditions.

In the fresh sample, we have determined the average composition of the films with the ratio of the measured XPS peak areas associated with each atom, following standard procedures^28^ and using the atomic sensitivities previously calibrated for the employed spectrometer.^29^ Assuming that the films are strictly homogeneous within the escape depth of the electrons, the ratio of the intensities of two atoms core-level peaks is related to the atomic density ratio (*X*_A_/*X*_B_) by *X*_A_/*X*_B_ = *A* × *I*_A_ /*I*_B_ where *A* = 1/*S*_A_/1/*S*_B_, with *S*_A_ and *S*_B_ being the atomic sensitive factor determined for the pure chemical elements for the specific electron analyzer used^30^: *S*_I_ = 6.0 and *S*_Pb_ = 6.7. The resulting atomic ratio composition I/Pb is 2.04:1, which is in perfect agreement with its chemical composition, thus confirming the validity of the used atomic sensitivity factor measured for the equipment.

**Figure 3:**
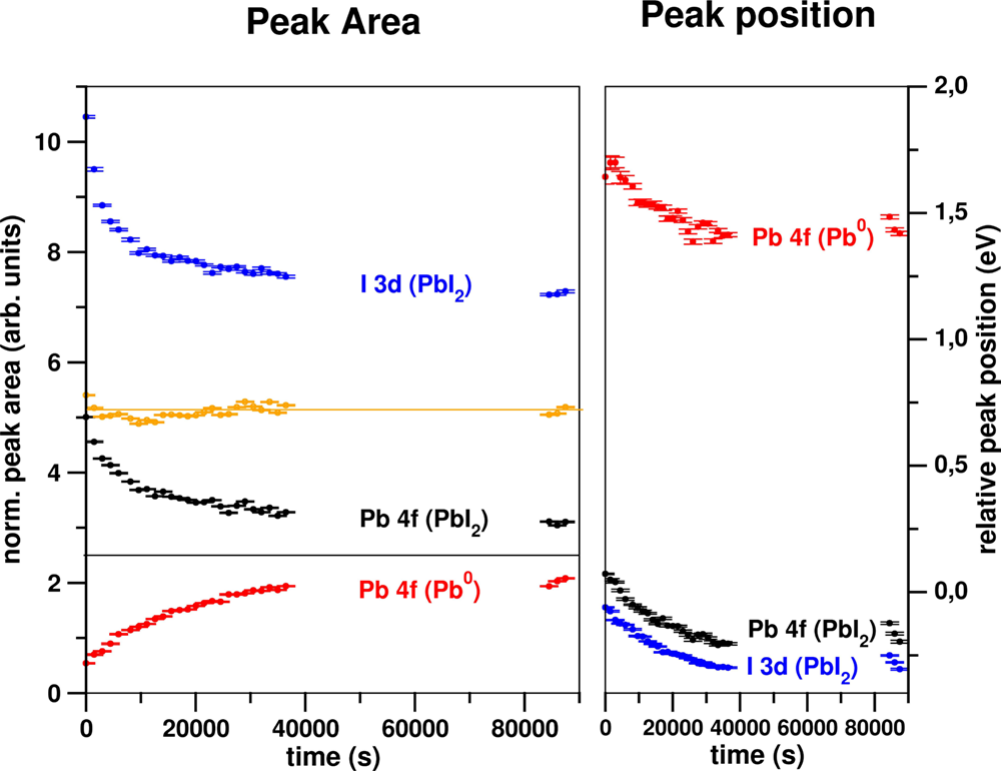
Measured I 3d and Pb 4f peak areas (on the left) and their relative peak position (on the right). Black and blue dots are Pb 4f and I 3d, respectively, associated with PbI_2_ and in yellow dots the resulting ratio area between them; In red, the peak associated with Pb-metal.

Figure 3 shows the atomic ratio deduced from the peak areas corrected by the atomic sensibility factor (left panel) and their peak position of the XPS peaks mentioned above (right panel) as a function of time. The initial PbI_2_ powder evolves with time toward a mixture of PbI_2_ and Pb-metal. After 1 day left PbI_2_ under UHV conditions it seems that the sample becomes stable, arriving to a final stationary point with a PbI_2_: Pb-metal ratio of 1.3:1. The evolution also reflects a clear decrease in the I 3d intensity. The orange line in the left panel of Figure 3 shows the sum of Pb atoms present in both forms: PbI_2_ and Pb-metal. It can be observed that the total amount of Pb atoms remains constant, indicating the direct transformation from PbI_2_ to Pb-metal with time; such a transformation is directly related with the decrease of the total amount of iodine (blue line in the left panel). Thus, decomposition of the sample with evaporation of I atoms seems to be responsible for such kinetics, i.e., I vacancies are created near the surface. The electronic characterization of PbI_2_ must be performed on the sample recently introduced into the UHV to avoid spurious results due to PbI_2_ decomposition; the experiments here below were made within an 8 h period from the sample introduction under the UHV environment.

**Figure 4:**
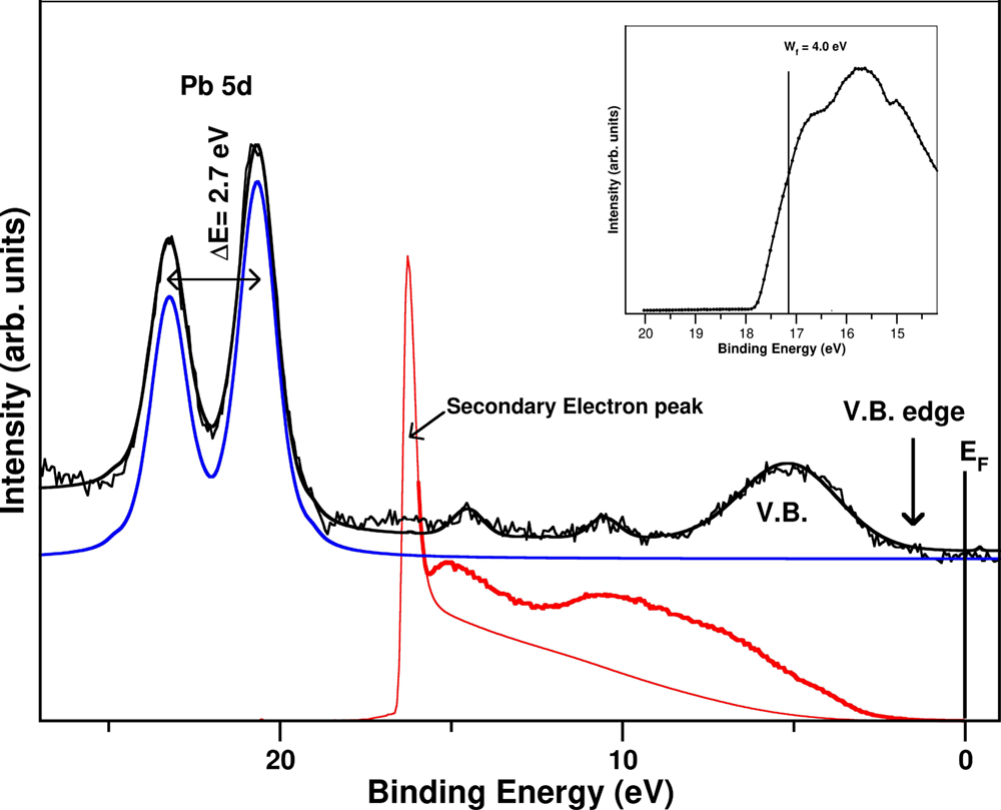
Measured UPS (red bold line) and XPS (black bold line) spectra on fresh PbI_2_ powder sample at the Fermi level proximity. In blue, the split Pb 5d XPS peak. In the UPS spectrum, the SECO intensity is shown by a thin red line. Figure inset shows the HeI-SECO spectra, which gives the measured *W*_f_ for the fresh PbI_2_.

The main electronic properties of a material are usually related to the electron valence band (VB). Thus, the determination of the electronic structure at a low BE, i.e., in the Fermi level proximity, is very important. This region was measured by both XPS and ultraviolet photoemission spectroscopy (UPS) experiments. In Figure 4, we show the measured XPS and UPS spectra close to the Fermi level for a fresh sample. UPS gives the electron orbital/band structure in the VB region, and it is the main technique used to characterize the low BE region. However, the UPS spectra are very dependent on the final state of the exited electronic structure,^31^ while the higher energy used in the XPS measurement makes it less dependent on the final excited state. In addition, in UPS, the photoemitted electron from the VB orbital is placed over a background signal coming from the inelastic scattered electrons (red thin line in Figure 4). All these facts makes the measured XPS spectrum a more suitable tool for the study of the orbital/bands at the VB region in this sample. Despite this, both XPS and UPS data give the same orbital position but UPS having less-resolved peaks. The difference in the intensity between the UPS and XPS in the coincident measure region is explained by the different atomic and orbital factor sensibilities of both techniques.

The XPS spectrum shows very clearly the PbI_2_ VB at 4.3 eV and the Pb 5d placed at 20.3 eV and its split spin–orbit at 23.0 eV, whose position and split correspond to PbI_2_. Other two very weak contributions appear at 14.4 and 10.1 eV.

The energy region close to the Fermi level has no intensity as it corresponds to a semiconductor material. The appearance of intensity defines the VB edge position which determines the semiconductor character of the material. Although the low electron density close to the Fermi level makes ot difficult to obtain the edge position with high precision, the combination of XPS and UPS (HeI and HeII) experiments allows to determine that the VB edge with respect to the Fermi level is located at 1.6 eV. This result is coherent with the band gap found in the literature for PbI_2_^32,33^ where they give an indirect band gap of 2.6 eV in PbI_2_ solid, but when it is on a monolayer, the nature of the band changes to having a direct band gap of 3.0 eV and an indirect band gap of 2.6 eV.^33^ Thus, the Fermi level should be placed close to the band gap half, giving semiconductor character for PbI_2_. The band gap energy is within the visible light energy range, making this material very interesting for optical detector materials.

In order to put this in absolute energy scale with respect to the vacuum level, we need to correct them by the PbI_2_ work function. The work function (*W*_f_) corresponds to the difference in energy between the Fermi level and the vacuum level (see the inset in Figure 4). The sample *W*_f_ was inferred from the secondary electron cutoff (SECO) spectra recorded with a DC sample bias of −10.0 V.^34^ It is experimentally derived by subtracting the cutoff photoemission energy (the edge placed at the highest absolute BE of the UPS spectra) from the photon energy (21.22 eV for the used He–I excitation photon energy).^34,35^ With the aforementioned UPS setup, the cutoff can be easily measured owing to the fact that electrons coming from the sample surface have an extra kinetic energy of 10 eV. That makes it possible for them to easily reach the electron analyzer and appear away from the secondary electrons coming from the electron analyzer.

The measured *W*_f_ in the fresh sample is 4.0 eV, in very good agreement with the values present in the literature that are in the range of 4–5 eV, depending on the sample preparation.^36^ In the aged sample, the already demonstrated presence of Pb-metal shifts the work function up to 4.4 eV, also in agreement with the values given in the literature.^27,37^ The simulations shown in section 2 provide further insight in this aspect.

### Photoactivity and Relaxation Kinetics

We have already pointed out that PbI_2_ has a band gap within the visible energy region, making it very interesting for photo-optical applications. Thus, Wei et al.^1^ showed that PbI_2_ single crystal exhibits a stable switchable photocurrent response to visible light photons (450 nm excitation at 5 mW cm^–2^) with reproducible ON/OFF ratios under 5 V bias. This behavior paves the way for PbI_2_ toward optical devices in advanced technologies such as environmental monitoring, imaging, and medical or industrial sensors.

**Figure 5:**
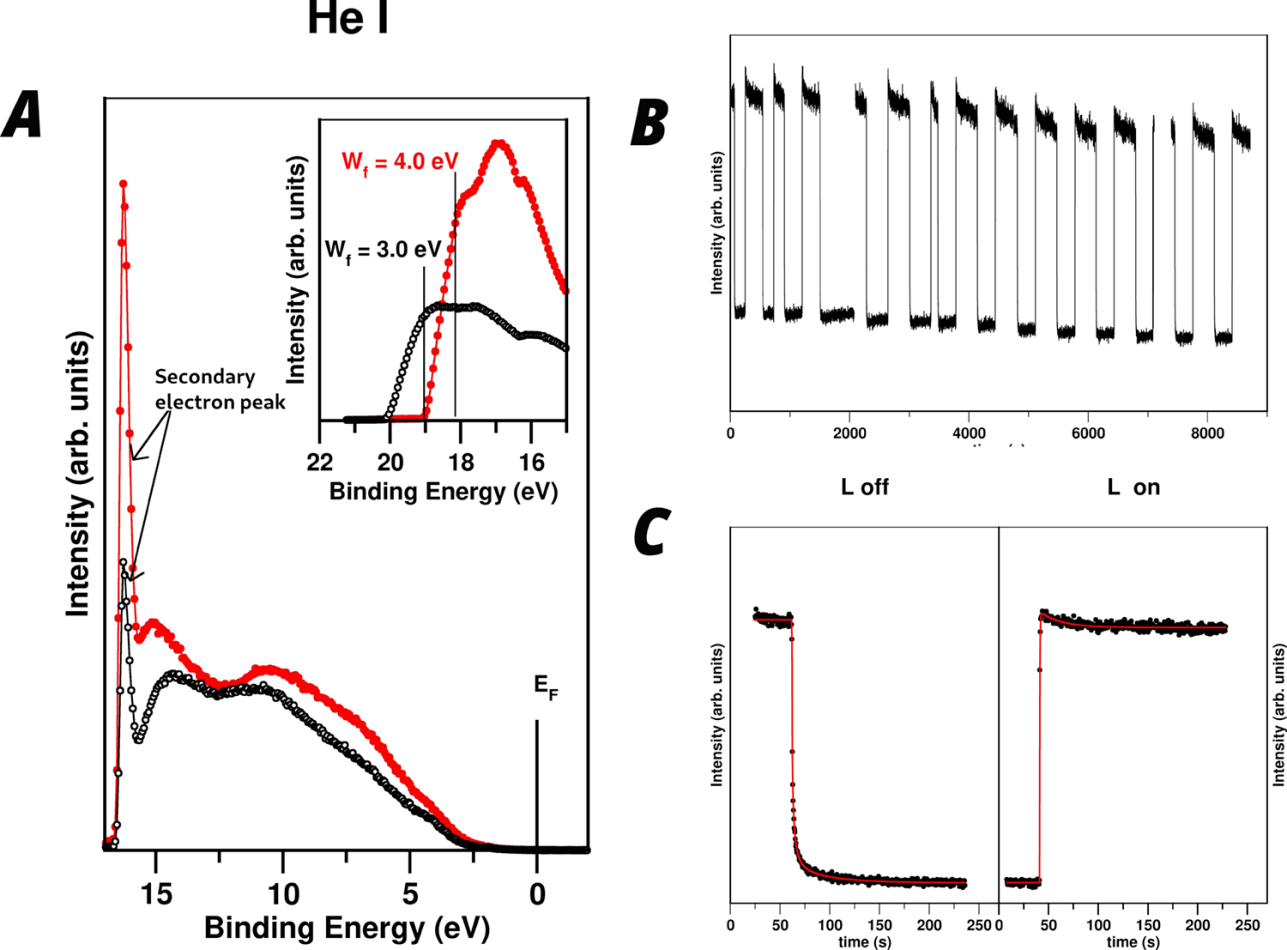
(A) Variation of the UPS spectra with light on (filled circles) and light off (holed circles) in the fresh sample. Figure inset shows the HeI-SECO spectra, which gives the observed change in *W*_f_ with light on/off. (B) Variation of intensity of the HeI-secondary electron peak spectrum measured at a fixed energy with time when light is turned on/off. (C) Details of the kinetics of the transient when light is turned off/on. The red lines correspond to the stretched exponential kinetic fit.

Interestingly, in this study, we have found that the photosensitivity of PbI_2_ goes even further. Indeed, it exhibits big changes in the electron bands close to the Fermi level when it is lightened with visible light, showing extremely long relaxation times. We have investigated the origin of these transient photocurrent processes in-depth. To do this, we have measured the dependence with time of the UPS spectra of PbI_2_ while switching on or off the light illuminating the sample. The HeI kinetic measurements have been carried out recording at a fixed BE in a value where the intensity has a clearly observable change when the light is turned on/off in the previously obtained UPS spectra. In most cases, this chosen energy position is close to the secondary electron peak of the UPS spectra. As secondary electrons are photoemitted electrons that have suffered inelastic processes, they can be considered proportional to a transient in the photocurrent. Figure 5A shows the UPS spectra of a fresh PbI_2_ powder sample irradiated with visible light. We can observe a change in intensity when the light is turned on/off. The inset of Figure 5A shows that the work function decreases by 1.0 eV when the light is off. This behavior is quite surprising because although the formation of electron–hole exciton pairs upon illumination in semiconductors is well-known, no changes are observed in photoelectron spectroscopy measurements due to its short lifetime. In all, three main features can be pointed out in the UPS spectra changes with light:

1. When light is on, PbI_2_ shows a peak shift and higher intensity in the VB in the HeI spectra (see Figure 5A).
2. The kinetics of relaxation is in all cases very slow, being of minutes order (see Figure 5B,C).
3. The transient photocurrent is a perfect reversible and stable phenomenon over time (see Figure 5B).

**Figure 6:**
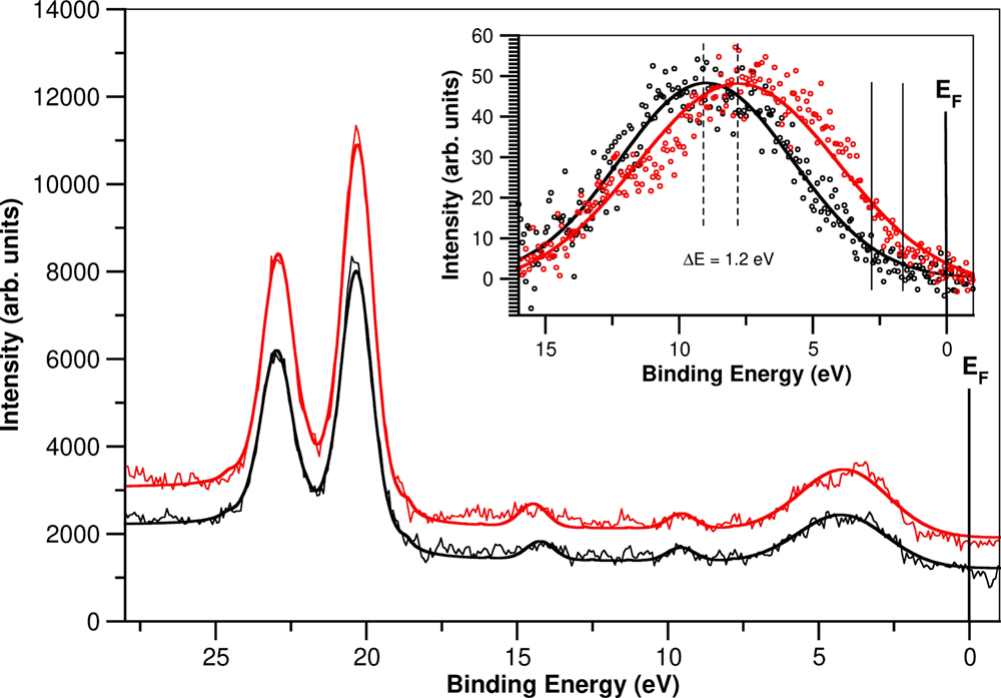
PbI_2_ VB region measured with XPS with and without visible light. The inset shows the changes observed in UPS He–II with light after exponential background removal and Gaussian peak fit (see also Figure S2 in the Supporting Information). Red and black lines correspond to the spectra measured with visible light on and off, respectively.

Furthermore, under illumination, we also observe a shift of the BE associated with both I 3d and Pb 4f core levels toward lower energies in 0.1 eV in a fresh sample This direction of the shift indicates a p-type SPV of the majority carriers for the PbI_2_ powder. The VB shifts to a closer energy to the Fermi level when the samples are illuminated (Figure 6), and *W*_f_ increases by 1.0 eV also when the samples are illuminated (Figure 5A). In the same direction, there is a change in the VB edge position with light. Under illumination, the VB edge shifts 1.2 ± 0.14 eV toward lower BE (shown as thin solid lines in Figure 6). (The VB edge is calculated as the peak position minus 2 times the σ for a Gaussian peak.)

This effect is less evident in the VB region measured with XPS because its higher energy makes it less surface sensitive.

All of these facts indicate that the PbI_2_ samples have a depletion band bending at the surface. Some authors relate this band bending to the presence of a surface photovoltage (SPV). With the SPV scheme, the observed UPS changes can be explained as light-assisted electron promotion to the conduction band (CB) accumulating charge close to the surface and driving a flat-band scheme. Furthermore, the SPV implies that there is an electric field on the sample surface. Many researchers have proposed the existence of various defects formed on the surfaces of powdered grains, such as vacancies and interstitial defects. It has also been shown, with calculations of the electronic structure, that various defects may generate different electronic trap states around both the VB and CB.^38^ These defects, whose presence is reflected in the intensity decay of the XPS I 3d peak (Figure 3), are at the origin of the SPV phenomenon.

We also studied the dependence of this effect on the wavelength of the incident visible light. The UPS changes are observed with blue and green light but not with red light. Considering the energy of the light in these colors (red: 1.74 eV; green: 2.34 eV and blue: 2.74 eV), we confirm that the band gap between the valence and the exited electron arriving level is in the 1.7–2.4 eV range. This range is within the order of magnitude of the values found in the literature for the energy gap in this compound, which is around 2.4–2.6 eV.^32,33^ Thus, the maximum observable band bending is given by the difference between the band gap (≃ 2.6 eV) and the VB edge position (1.6 eV) which agrees with the shift observed in *W*_f_ with light (see Figure 6).

Differences in the peak shift values of XPS and UPS are related to the different penetration depth of both techniques.^39^ During the spectroscopy measurements, absorption occurs at a certain sample depth, but the SPV signal (shift in BE associated with a change of band bending) results only from the contribution of the surface space charge region associated with the SPV phenomenon. It is worth mentioning that band bending is not occurring at the whole sample scale because the sample grain thickness is larger than the depletion width and the penetration depth of the spectroscopy used technique. The penetration depth of each electron spectroscope is given by the electron mean free path that depends on the electron kinetic energy. The dependence of the inelastic electron mean free path with energy is mainly independent of the material and is given by the so-called universal curve. In this curve, the electron free path depends on the electron kinetic energy and is independent of the material. This universal curve of the atomic mean free path has a minimum at 40 eV and increases at both lower and higher energies. Thus, the HeI (21.2 eV) measurements correspond to the topmost surface layers (3–5 Å approx.), the HeII (40.8 eV) which has a higher penetration deep (6–8 Å approx.), and XPS (1253.6 eV) which has a typical measurement deep range of 50–100 Å. Thus, meanwhile the UPS reflects the SPV band bending of the two topmost layers of the surface sample, the XPS corresponds to the average SPV band bending of the 50 topmost surface layers, resulting in smaller SPV peak shifts than the *W*_f_ shift observed by UPS HeI. Thus, the SPV signal results only from the contribution of the surface space charge region, which has an extension in the 10–50 Å range. The other effect that depends on the source energy used in each technique is the change in intensity when the light is on/off. An increase in the intensity observed in the VB region of the HeI spectra is observed when light is on. This intensity change is not observed in the HeII valence or in the XPS core-level spectra. This fact could be related to an anomalous change in the effective section in the proximity of the white light activation high edge.

In order to have a more complete image of the origin behind the observed transient photocurrent effect, we have determined the kinetics of the relaxation process with time. Figure 5C shows a fit of the relaxation kinetics corresponding to the HeI signal when the light is turned on/off. Relaxation kinetics arise as the difference in decay times between photocurrent and electron spectroscopy measurements. The measured relaxation time is of tens of seconds, which is immeasurably longer that the exciton lifetime. HeI photocurrent kinetics do not fit to a simple exponential equation. Thus, more adequate models have been used.^40,41^ The best fit is obtained with a stretched exponential decay.^40^ The photocurrent transient decay is observed in PbI_2_ when light-off follows a stretched exponential decay, known as the Kohlrausch–Williams–Watts stretched exponential function:
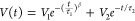
1where 0 < β < 1. The first term represents the stretched exponential decay. Compared to a simple exponential decay (where β = 1), this exponential decay is faster in the initial decay time but slower in the final part of the decay. Historically, Kohlrausch used this stretched exponential function to explain the decay of the residual charge in a glass Leyden jar in experiments related to the relaxation of complex electronic and molecular systems. In general, this function is applicable when a process deviates from simple first-order kinetics, wherein the decay rate is not constant but decreases with time as *t*^1−β^.^30,42^ The stretched exponential decay is also observed in electronic transport in semiconductors, nanocrystalline, and conjugated polymers owing to molecular/crystal relaxation, trap states, or disorder properties.^43^ The equivalent stretched exponential equation with light-on is
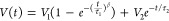
2

The curve fit is shown in Figure 5C (red line), and the obtained parameters are given in Table 1. The resulting stretched exponential decay is  both for light-on and light-off processes, which is commonly attributed to a two-dimensional charge-transport process. This result is consistent with the 2D structure obtained for PbI_2_, where the planes in the Pb–I octahedron act as 2D containers for charge carriers confining the conduction mechanism. At the same time, it suggests the presence of various trap states at the interface between planes or at the surface of the power grains, where the electron-trapping process is accompanied by a two-dimensional electron transport among the trap states.^44^ At this point, it is worth mentioning that in the bulk material, the cations and anions produce a charge balance leading to electric-charge neutrality.^45^ In contrast, on the grain surface, there will be many unpaired charges, even in the absence of vacancies or interstitial defects. In addition, the presence of vacancies or interstitial defects generates more defect states and surface states with different energy levels and degrees of localization. The electric field resulting from charge accumulation or light illumination may disturb this electric-charge balance, which may also temporarily or permanently displace the ions from their original positions. The opposite situation may also occur wherein the misplaced ions are relocated to their ideal position in the crystal structure. Bertoluzzi et al.^46^ and Pockett et al.^47,48^ have demonstrated the possibility of photoinduced ionic migration based on their observations of long-lived decay in oxidative chemical vapor deposition experiments.

**Table 1:** PbI_2_ Fit Parameters of the Kinetic of Relaxation

	χ^2^	τ_1_ (s)	β	τ_2_ (s)	*D*_carriers_ (mm)
light-off	1.11	0.68	0.47	38.30	0.19
light-on	1.02	0.40	0.50	31.13	0.85

The τ_1_ parameter refers to the mobility of the minority carriers when light is on (eq 1), thus the electrons, and to the mobility of the majority carriers when light is off (eq 2), thus the holes. The diffusion coefficient for charge carriers can be obtained by the Einstein relation according to
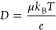
3and the diffusion length of eqs 1 and 2 can be written as
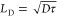
4Taking into account eq 3 and considering the value of the mobility of holes and electrons given in the literature for PbI_2_^49^ (μ = 1.94 cm^2^ V^–1^ s^–1^ and μ = 69.52 cm^2^ V^–1^ s^–1^, respectively), we can obtain the diffusion coefficient for holes and electrons (*D* = 0.0502 cm^2^/s and *D* = 1.7988 cm^2^/s, respectively). From these values and knowing the relaxation times of electrons and holes, the diffusion length can be obtained according to eq 4.^50^ The resulting diffusion lengths with light on/off are given in Table 1, showing huge diffusion lengths (close to 1 mm).

With the theoretical SPV models used, the τ_1_ parameter is related to the charge diffusion rate (τ_1_eq 2). Therefore, it can be concluded that at short times in the transient of light-on, the system will be dominated by a self-decelerating electron diffusion term while the light-off transient can be modeled by a diffusion-type term for holes (τ_1_eq 1). At long times (τ_2_ in eqs 1 and 2), the kinetics is dominated by the relaxation time of the impurity levels that can be located in the middle of the gap of PbI_2_.^44^ The extremely slow relaxation process indicates that the mechanism involves charge transfer through a discrete energy level placed within the band gap, whose symmetry is such that the transition has a small probability. We discuss the origin of this level later.

### Theoretical Study

**Figure 7:**
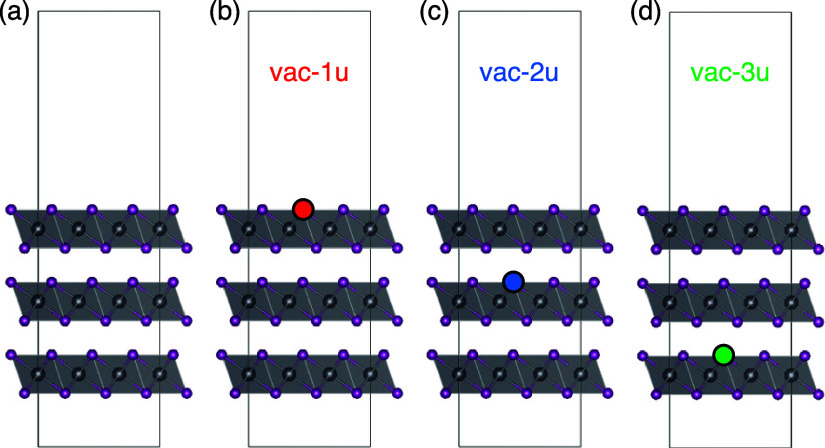
Slab models considered (a) without vacancy; (b) with vacancy in the first layer in the upper I atom: vac-1u; (c) with vacancy in the second layer in the upper I atom: vac-2u; (d) with vacancy in the third layer in the upper I atom: vac-3u.

Further insight into the electronic properties of PbI_2_ and its behavior when it is lightened with visible light has been obtained by means of density functional theory (DFT) calculations. For a complete comprehension of the electronic structure, we have considered bulk and slab models representing the material and the surface, respectively. In both bulk and surface, we have also considered situations with vacancies, where one I atom is removed. In the case of the bulk, the I vacancy has been created in a cell with 3 × 3 × 3 unit cells. In the case of the surface models, three possible I vacancies were taken into account (see Figure 7). In these slab models, the vacancy is created in the upper I atom of each PbI_2_ layer. In those cases without vacancy, we have first optimized the geometry and then computed the electronic structure. In those models including a vacancy, we did not reoptimize the geometry. As an approximation, we computed the electronic structure using the previously optimized geometry, just removing the I atom.

**Figure 8:**
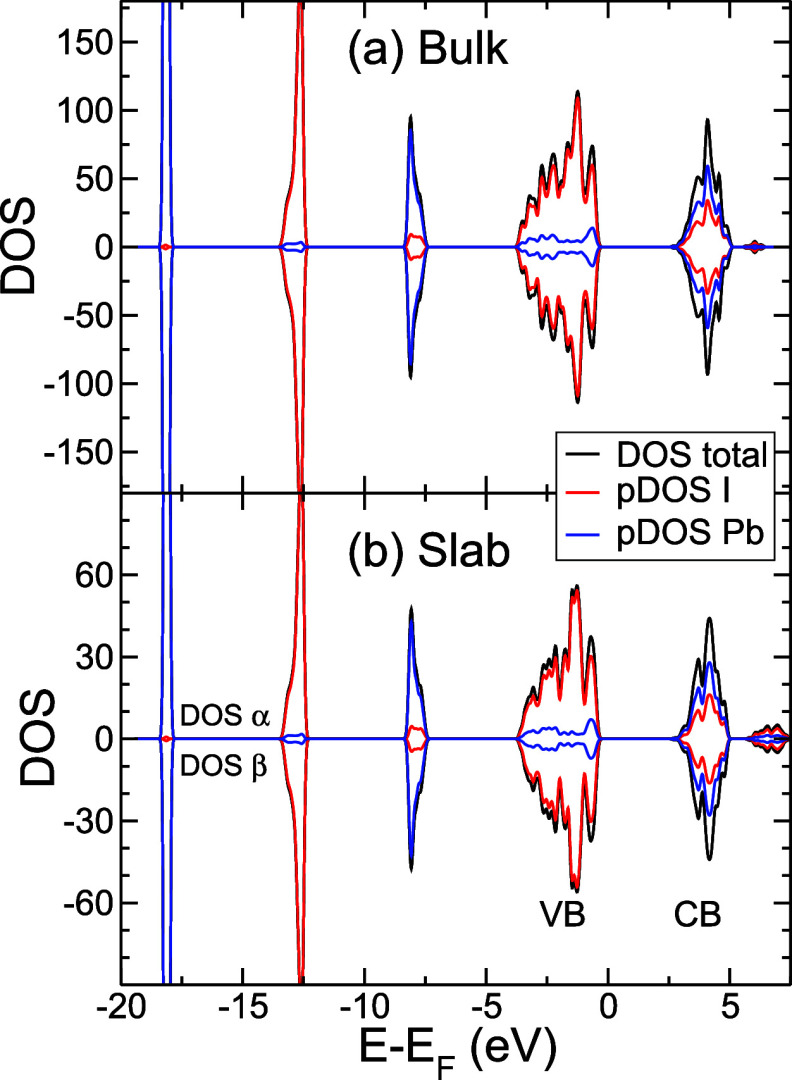
Density of states. (a) 3 × 3 × 3 Bulk model and (b) three-layer slab model, both without vacancy, computed at the HSE level over the OPTPBE geometry. α and β electrons are represented by positive and negative signs, respectively. black line: total DOS, red line: DOS projected on I atoms, and blue line: DOS projected on Pb atoms.

The optimized geometry for the bulk has been already discussed, and we here focus on the electronic structure. We start analyzing the computed density of states (DOS) and the DOS projected on the two type of atoms, both in the bulk and in the surface without vacancies (see Figure 8). The difference between both is negligible, confirming that it is a layered material, where the electronic properties of the 2D PbI_2_ planes on the surface are reflected in the bulk. The VB is mainly composed of I orbitals (5s and 5p), and in the CB, both I (5s and 5p) and Pb (6s and 6p) orbitals contribute.^51−54^

The Pb and I outer s and p orbitals forming the VB are characterized by a relative low photo ionization cross-section giving an UPS spectrum of quite low intensity (i.e., Pb 6s and 6p has a photoionization cross-section 2–3 orders of magnitude lower than the Pb 5d).^55,56^ Furthermore, photoionization is also affected by photoemission final states, resulting in an UPS spectrum in the vicinity of the Fermi level with lower definition than the XPS one.

**Figure 9:**
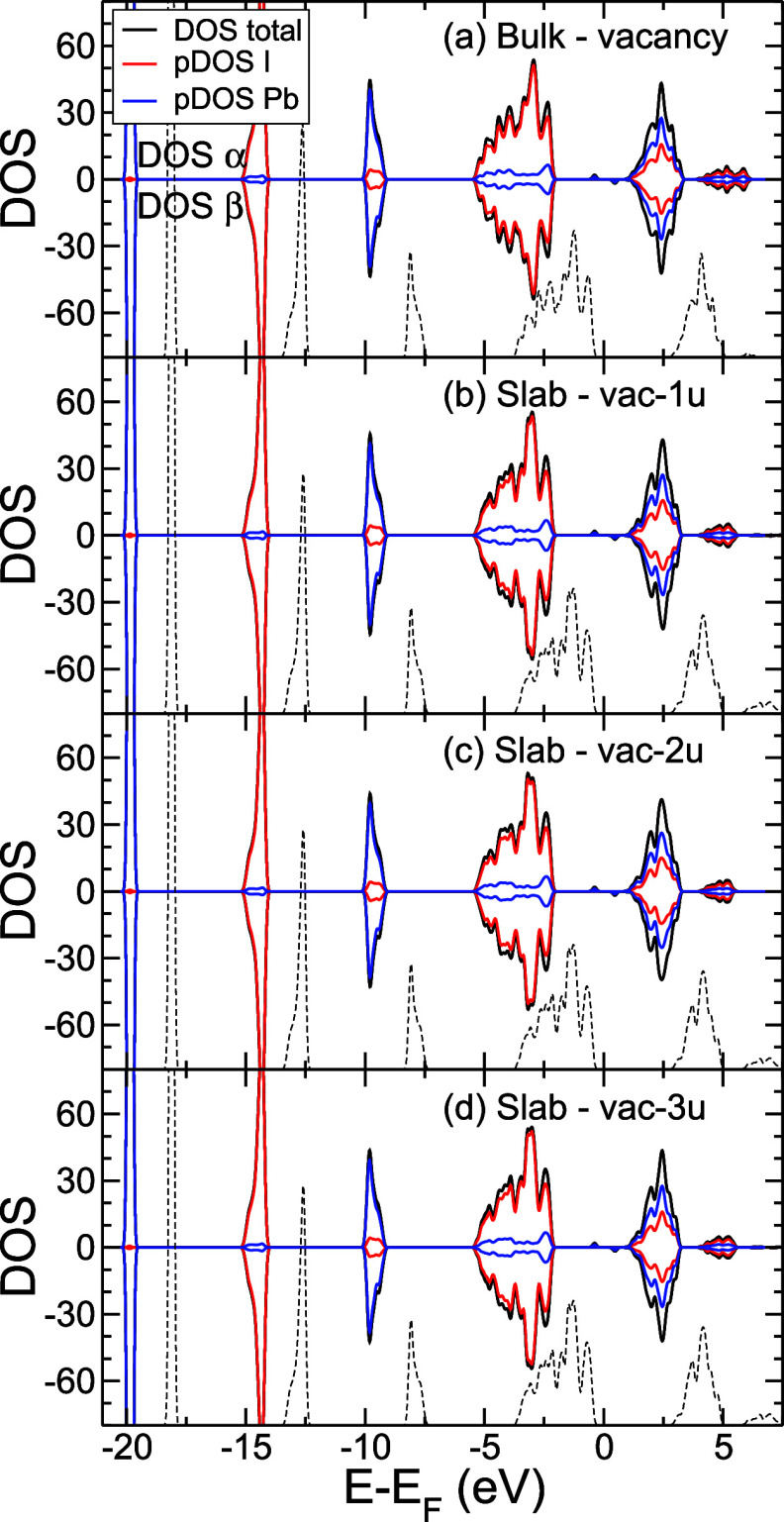
DOS. (a) 3 × 3 × 3 Bulk model with vacancy, (b) slab model with vacancy vac-1u, (c) slab model with vacancy vac-2u, and (d) slab model with vacancy vac-3u (see Figure 7), computed at the HSE level over the OPTPBE geometry. α and β electrons are represented by positive and negative signs, respectively. black line: total DOS, red line: DOS projected on I atoms, and blue line: DOS projected on Pb atoms. Dashed line corresponds to the corresponding model without vacancy for comparison in (a) bulk without vacancy and (b, c, d) slab without vacancy.

Figure 9 shows the effect of introducing I vacancies in the bulk and slab model. The main difference is a shift, lowering the density of states of the VB and the CB in about 2 eV with respect to the Fermi level. The reason behind such a change is the appearance of an occupied state in the middle of the gap, associated with the subtracted I atom, thus changing *E*_F_. This effect is similarly observed in both the bulk and the surface.

Further characterization of the effect of the vacancy is given in the Supporting Information (see Figure S3), where we show a zoom with the comparison of the DOS of α and β electrons in the bulk near the Fermi level (see also Figure 9). An occupied state associated with the vacancy is located 0.39 eV below calculated *E*_F_ and an empty state 0.47 eV above calculated *E*_F_. (The value of Fermi level obtained by the DFT calculations is one of the Kohn–Sham eigenvalues of the calculated system, and it does not correspond to the value of Fermi level in the experiment) The states are quite localized on the impurity and present the participation of both I and Pb atoms.

**Figure 10:**
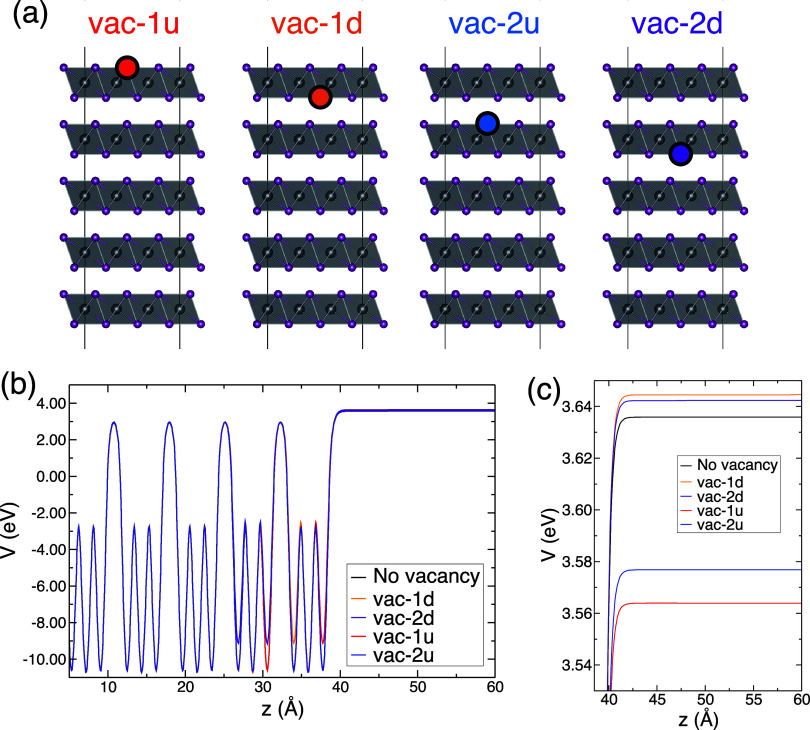
(a) Four possible vacancies in a five-layer slab model: vacancy in the first or second layer with the I atom removed from the up or from the down. (b) Averaged potential as a function of *z* computed at the OPTPBE level of theory. (c) Zoom on the potential - the value of the potential directly indicates *W*_f_. The Fermi level in each model lies at the following values. Model without vacancy: −2.41 eV, vac-1d: −0.87 eV, vac-2d: −0.89 eV, vac-1u: −0.89 eV, and vac-2u: −0.90 eV. See also Figure S4 in the Supporting Information for the effect of the dipole correction at long distances from the surface.

The effect of the vacancy also affects the work function of the PbI_2_ surface. We analyze such an effect with the computed results shown in Figure 10. After the analysis of the electronic structure using three PbI_2_ layers, in this case, we have increased the slab model considering five layers to correctly describe the effect at long distances away from the surface. The energetic properties are directly computed with the OPTPBE functional, as given from the geometry optimization of the model without vacancy. We present the averaged potential as a function of the coordinate perpendicular to a surface with and without vacancies; considering the impurity in different positions, vacancies in the two outermost layers with the I removed from up and down from the layer. The long-range value of the potential referred to the Fermi level directly indicates *W*_f_. Interestingly, the location of the impurities changes the *W*_f_ value differently, increasing or decreasing it. Such changes are based on the fact that the impurity creates an occupied state with a rather localized excess of charge, leading to a surface dipole. The direction of such a dipole depends on the position of the vacancy and consequently increases or decreases the potential. If an iodine atom is removed from the outermost PbI_2_ layer, the potential at long distances decreases (increases) with respect to that of the nondefected surface if the impurity is found above (below) the Pb atom, i.e., an ejected electron suffers a dipole pointing in opposite directions. Notice that if the impurity is created in an internal layer of PbI_2_, the effect is less marked since there is a screening of the dipole by the outer layers; thus, the potential changes to a lesser extent. Evaporation of the outermost I atom is expected to occur easily; the computed energy required to extract one I atom, without considering any geometrical relaxation and producing a vacancy in the upper outermost layer, the most stable case, is 3.78 eV. This situation corresponds precisely to the largest change in *W*_*f*_, with a decrease of 0.07 eV.

**Figure 11:**
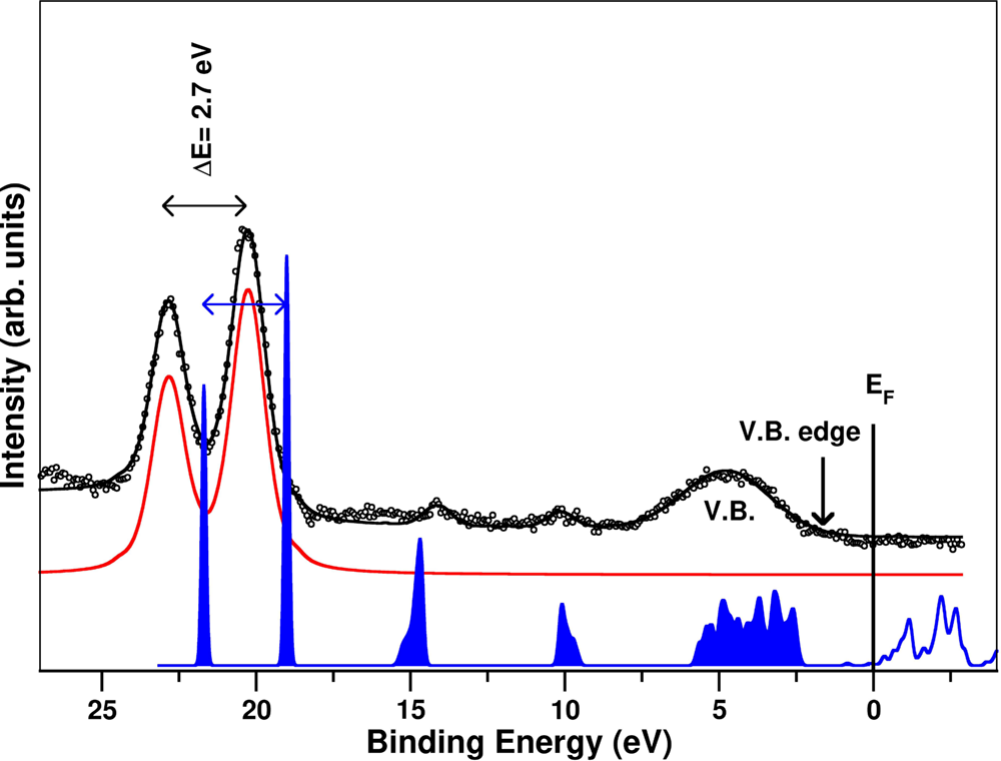
Measured XPS spectrum at a low BE region compared with the calculated DOS. The red line corresponds to the Pb 5d orbital fit. The calculated DOS is shifted 0.4 eV compared with the experimental data. Thus, the Fermi level for the calculation is set close to the LUMO.

We finally present in Figure 11 a direct comparison of the computed DOS with the experimental XPS measured at a low BE, i.e., close to the Fermi level. As we already pointed out, the UPS in this system has lower resolution due to different factors. In addition, photoionization cross sections for s and p orbitals in Pb and I atoms are of the same order of magnitude, and the XPS can directly be compared to the calculated DOS. The Pb 5d atomic orbital has a significant higher photoionization cross-section (factor of 400 approx.) than the observed bands associated with s and p orbitals, and thus, it clearly stands out in the spectrum with a BE of 20.3 eV; its position in the spectrum is used as a crosscheck of the position of the Fermi level for the calculated DOS. We have chosen for comparison the bulk model with a vacancy since, as we already pointed out, this is a situation that occurs frequently. We obtain a very good agreement between the simulations and the measurements. Notice that this calculation includes the spin–orbit coupling, and it perfectly reproduces the splitting of the Pb 5d orbital, Δ*E* = 2.7 eV, in both the experiment and the theory. The simulations also reproduce the small features measured at 10 and 15 eV of BE.

### Photoactivation Mechanism

**Figure 12:**
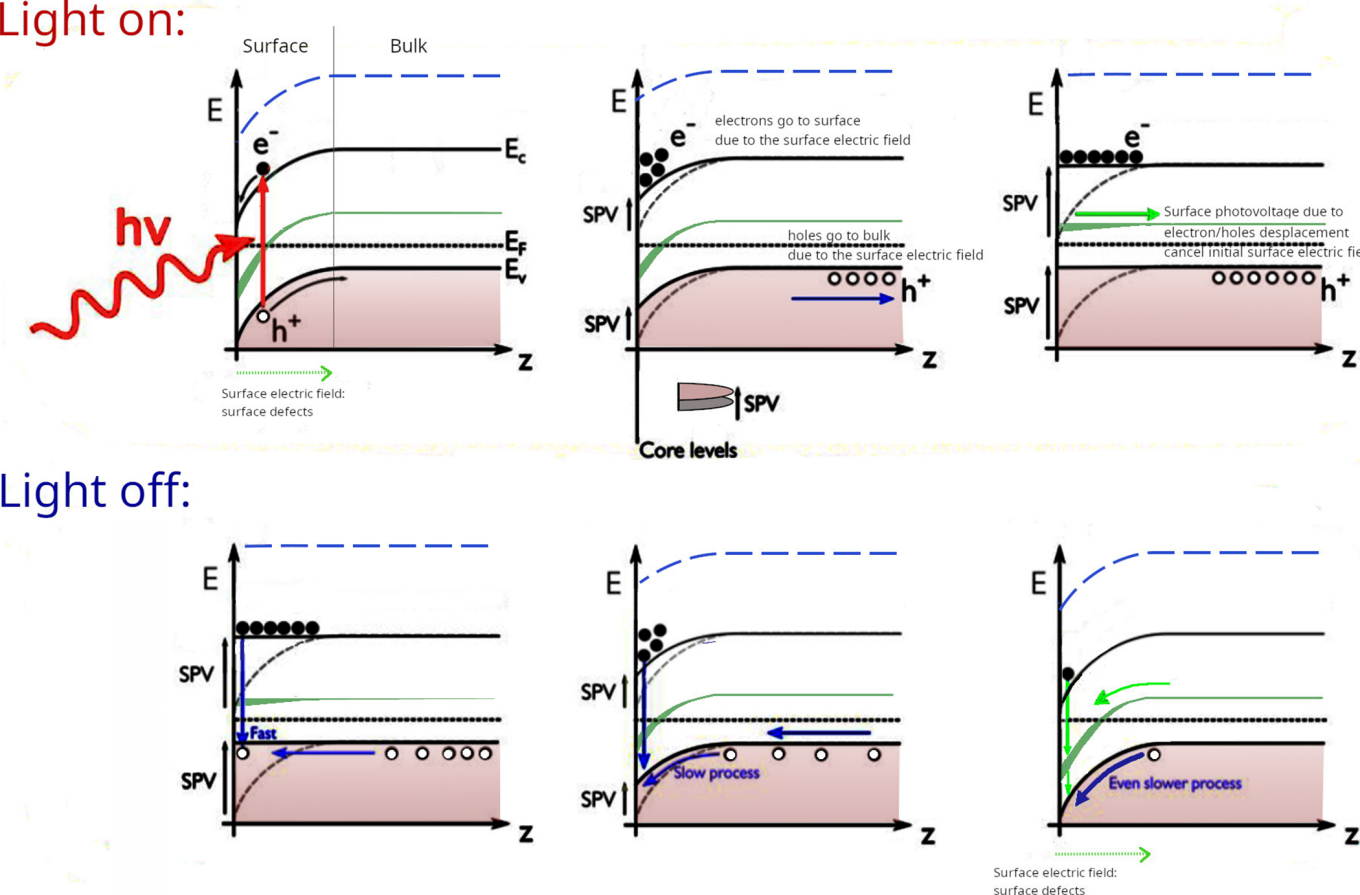
Sketch of the electron and hole excitation/relaxation process with light on/off, respectively.

Considering all of the above measurements and simulations, we propose a photoactivation mechanism that explains all observed phenomena (see sketch in Figure 12). In the absence of light, PbI_2_ presents a surface electric field. The calculations have shown that its origin is in the presence of iodine vacancies, and it induces charge transfer at the surface interface. Thus, there is a natural self-doping of the material on the surface that modifies the position of the bands with respect to the Fermi level and changes *W*_f_. As a result, there is a band bending on the surface. Such self-doping, originated by the presence of vacancies, is also supported by the fact that there is no evidence of the presence of other atoms that could act as doping atoms. The direction of the electronic band and the shifts observed in the *W*_f_ indicate that it is a p-type SPV (p-SPV).

Upon sample illumination with visible light, a homogeneous shift of the photoemission peaks is observed, while the characteristic substrate peaks and the Ag colloid used as powder glue remain in the same position. This effect can be understood as a SPV effect as depicted in the Figure 12. Once photons are absorbed, electron–hole pairs (excitons) are generated, and the electrons and holes are separated; because of the downward band bending occurring at the surface, the electron moves toward the surface (see Figure 12). In a similar way, the hole moves toward the sample bulk. It is important to note that this electron hole segregation, naturally achieved in our case by the defects-generated electric field, is a fundamental process in the performance of any photovoltaic device. Moreover, to produce electric current, it is not only important to generate an electron–hole pair but evenly important is to separate them, allowing the independent collection of electrons and holes current that can be directed in an electric circuit.

As the photoexcitation process continues, the negative charge accumulates on the surface, while the holes migrate toward the bulk (see Figure 12 top middle panel). Such an accumulation of negative charge on the surface compensates the original positive charge created by the defects (iodide vacancies), thus decreasing the electric field on the surface and lowering the surface band bending. Therefore, this flow of charge carriers is a self-decelerating process because the driving force is band bending and such bending is reduced by the process itself. Such reduction of band bending is evidenced as a shift toward lower BE of the photoemission peaks, a behavior known as p-SPV, as detailed above.

While the light is on, the process continues up to a saturation state in which the accumulated electrons on the surface fully compensate the positive charge originating from the I^–^ vacancies, thus annihilating the electric field. Consequently, surface band bending decreases until a flat-band situation is reached (see the top-right panel in Figure 12). Clearly, the maximum photovoltage signal that can be observed depends on the PbI_2_ layer band gap and on the position of the Fermi level. The maximum measured SPV shift for the core level in our case is 0.15 eV, and the shift observed in *W*_f_ is 1.0 eV. Our results also evidence that PbI_2_ is an n-type semiconductor with a band gap close to 2.4 eV (energy associated with the wavelength of the light used for the photon-excitation), with the VB edge being placed at 1.6 eV with respect to the Fermi level. Thus, the band gap deflection is limited by the difference between them, in good agreement with the shift of 1.0 eV observed in both *W*_f_ and the VB edge peak position.

We theoretically calculated changes in *W*_f_ for several situations where the vacancy is found in the first two layers at different heights with respect to the surface (see Figure 10). The computed results are in good agreement with those found in the literature.^36^ Simulations show that the PbI_2_ layers behave independently and that vacancies can be easily formed at the surface. Thus, the intrinsic defects, I vacancies, are inevitably associated with a PbI_2_ monolayer and, in consequence, there is an atomic level within the PbI_2_ band gap associated with the defect.

Vacancy iodine defects generate in-gap defective levels, which appear mainly due to the unsaturated chemical bonds of the p orbitals of the exposed Pb atoms. The discrete defect level is sketched in Figure 12 in green. Such rich defective levels become reservoirs or sinks of electron/hole carriers directly influencing the electronic properties in the region of the defect in two aspects: (1) forming a Schottky-type interface in which the built-in potential facilitates the electron–hole separation and extends the carrier lifetime and (2) acting as the recombination centers due to the deep defective levels. To promote the efficiency by the Schottky effect, Chen et al.^36^ suggested that the kinetic is lead by a mechanism of defect recombination and defect suppression centers. The study of the kinetic relaxation with light on and light off shows that this process is confined in a 2D PbI_2_ layer.

Finally, when the light is off (bottom panels in Figure 12), recombination of the electron–hole pair occurs on the surface. At the beginning of the relaxation, when the bands are still flat, it is a fast process. However, when the band bending appears again, it slows down the relaxation process. In the recombination, the in-gap discrete levels, created due to the I vacancy, play a role, and the charge carriers flow through these levels. While relaxation occurs, it is an increasingly slower process, since the band bending is growingly pronounced and the flow of charges is continuously decreasing.

In summary, the photoactivity mechanism is previous to the photon-excitation, the PbI_2_ shows a band bending and a defect in-gap level; upon irradiation with visible light, the electrons are excited and the formed electron–hole pairs are physically separated by the SPV, transferring electrons toward the surface and holes toward the bulk; the result is that the electron migration compensates the surface-localized positive charge, and the bands become flat. The relaxation process goes in the opposite direction but, in this case, the electron relaxation passes through the defect in-gap level. This is precisely the kinetically limiting process, the relaxation bottleneck, making it extremely slow that it becomes an experimentally observable process. Note that in the material's photoactivity, the trap states are mainly generated by the huge amount of elemental Pb present on the surface, and thus it is reflected in the measurements. Usually, trap state concentrations are already relevant on the electronic properties when 1/10,000 atoms are affected, and in our case, we have a much higher amount of defect concentration. The mediation roll of the metallic lead in the lead iodide and its related organic–inorganic perovskites is still an open subject, but it seems to be related with the induced lattice relaxation.^57^

## Conclusions

We have experimentally and theoretically characterized the structure, electronic properties, and behavior upon visible light irradiation of PbI_2_. It is a layered easily exfoliating material that decomposes with time, even under UHV conditions, suffering sublimation of iodine atoms from the sample surface. Such defects create an accumulation of positive charge that generates a surface electric field which is responsible of SPV phenomena observed. Self-doping by vacancies has a great influence on the electronic properties, enhances light absorption, creates a channel for electron–hole transfer, and acts as preference molecular adsorption sites, characteristics that made PbI_2_ a 2D material with strong potential for photocatalysis purposes.

With our measurements, we have undoubtedly shown the presence of a reversible and stable transient photo current in PbI_2_, making this material very interesting also with potential applications in photodetectors. We have characterized the speed of response of lead-iodide detectors, where the induced pulse is primarily due to the motion of either electrons or holes. This enables the characterization of the transit effects of electrons and holes independently.

The complete electronic characterization of the response under visible light described here has allowed us to provide a complete scheme of the electronic configuration and mechanism that drives the transient photocurrent. Thus, this study shines a light on the behavior of perovskites that includes 2D PbI_2_ layers in their structure as key components in solar cells.

## Methods

### Experimental Section

All measurements reported in this paper were conducted on polycrystalline PbI_2_ powder purchased from Sigma-Aldrich (ref:900168, 99.999% purity).

Powder X-ray diffraction data were collected using a Siemens D5000 diffractometer equipped with a Cu anode for the structure determination of the samples. The diffractometer was configutred in the Bragg–Brentano setup with the sample rotating around the diffractometer ϕ – axis with a rotational speed of 4 Hz. The diffracted signal was recorded using a NaI(Tl) scintillator from Oxford Instruments combined with an automatic attenuator device^58^ to increase its dynamic detection range. The detector arm was equipped with a graphite monochromator in order to suppress the signal due to fluorescence and the *K*_β_ line. To select useful events, a tunable differential discriminator with a reconfigurable energy window was used with the amplified detector signal. The energy window was selected with the aid of a multichannel analyzer. The collected diffraction data corresponded to both Cu *K*_α_1,2__ lines; the contribution of the Cu *K*_α_2__ line was later removed from the recorded data by a mathematical procedure using a fit with penalized splines.^59^ The sample powders were milled in an Agatha mortar and deposited in a zero-background Si sample holder. The obtained diffraction pattern was indexed using the N-TREOR9 software^60^ integrated in the EXPO2014 package.^61^ The structure refinement consisted of the determination of the lead–iodine octahedral by direct methods followed by a final step Rietveld refinement.

All electron spectroscopy experiments reported in this paper have been carried out under UHV conditions in an experimental chamber with a base pressure of 2 × 10^–10^ mbar. An X-ray source with a Mg anode produces photons of energy *h*ν = 1253.6 eV from its *K*_α_ emission line that are used for XPS measurements of the atomic core levels. A He discharge lamp provides He–I (*h*ν = 21.2 eV) and He–II (*h*ν = 40.8 eV) photons allowing to explore the systems’ VB by means of UPS)measurements. For both types of experiments, the same hemispherical energy analyzer (LEYBOLD LHS10) has been used. Its pass energy was set to 50 eV for XPS to reach a resolution of 0.7 eV and to 5 eV for UPS yielding a final resolution of 0.1 eV. For the analysis of the XPS peaks, the contribution of the Mg *K*_α_ intrinsic line has been eliminated by deconvoluting them with an iterative Richardson–Lucy algorithm that was applied until reaching a maximum in the Shannon entropy.^62^ The Mg *K*_α_ satellites have also been removed with the help of an automated algorithm employing constrained penalized spline fitting.^59^ After this procedure Tougaard background^63^ was subtracted, and the peaks were fitted with a Doniach-Sunjic combination of Lorentzian and Gaussian functions.^64^

For photoemission measurements, PbI_2_ was used as supplied and glued directly to the Mo sample holder plate with a Ag liquid colloid to ensure good electrical conduction and to prevent that the sample powder falls. Thus, all core levels are referred to the Ag 3*d*_5/2_ state of the colloid, with a BE of 367.2 eV, which is weakly visible in the total XPS spectra.

The energies of the UPS spectra are referred with respect to the Fermi edge of a Ag(100) single crystal used as energy reference. The Ag single crystal was prepared in situ by cycles of Ar^+^ ion sputtering with ∼2 μA/cm^2^ sample current density and annealing at 900 K, until negligible contamination was detected on the surface and a sharp LEED pattern could be observed.

### Computational Details

All simulations were carried out in the frame of DFT, including periodic boundary conditions, and using the Vienna Ab initio Simulation Package (VASP).^65−68^ For geometry optimizations, we employed the OPTPBE^69,70^ functional, which includes weak interactions (i.e., van der Waals forces). The electronic structure was computed with the HSE06 hybrid functional^71,72^ over the geometries previously optimized. We computed bulk properties, sampling the reciprocal space with the Monkhorst–Pack scheme and using three points in each direction (3 × 3 × 3). For the slab models, which consist of three PbI_2_ layers and include 24 Å of vacuum, we kept the number of points along the periodic directions and used a single point along the direction perpendicular to the surface (3 × 3 × 1). In all cases, the electron density was expanded in a plane-wave basis with an energy cutoff of 350 eV, and the interaction between electrons and nuclei was simulated through projected augmented wave pseudopotentials, as downloaded from the VASP database. We imposed an energy convergence criterion of 10^–5^ eV for the self-consistent field. Structures were considered as optimized when all Hellmann–Feynman forces were lower than 0.01 eV/Å. In the slab model, the atoms in the first PbI_2_ layer have been allowed to relax, keeping the rest frozen with the geometry optimized for the bulk. In those models including a vacancy, we considered the geometry optimized without vacancy and was not reoptimized. The 1T phase of PbI_2_ is considered in all cases. To analyze the effect on the electronic structure of the vacancy, we kept the optimized geometry of the slab without vacancy, and we removed the corresponding atom. Over this structure, single-point energy calculations were performed to obtain the DOS. In all cases, the DOS has been computed applying a Gaussian smearing and using a width of σ = 0.1 eV. The effect of the density of vacancies in the electronic structure is given in the Supporting Information (see Figure S5).

## Supporting Information Available

XPS data for the commercial PbI_2_ sample at two different moments after the bottle opening; UPS measurement for fresh PbI_2_ sample with/without visible light illumination; and DOS near the Fermi level for the 3 × 3 × 3 bulk model including a vacancy (PDF)

## Notes

Crystallographic data for PbI_2_ has been deposited at the CCDC under deposition number 2363489.

## Abbreviations

UHV: ultra high vacuum
XPS: X-ray photovoltage
UPS: ultraviolet photon spectroscopy
SPV: surface photovoltage
DOS: density of state
KWW: Kohlrausch–Williams–Watts
HOIP: hibrid organic inorganic perovskite
TMD: transition-metal dichalcogenide
PAW: projected augmented wave
DFT: density functional theory
