# Supplementary material for: Disentangling the Optoelectronic Behavior of Lead Iodide Governed by Two-Dimensional Electron Confinement
Source: ACS Appl Mater Interfaces. 2024 Oct 15;16(42):57302–15. doi: 10.1021/acsami.4c10507 (PMC11503613; doi:10.1021/acsami.4c10507)
Supplement: Supplementary file 1 — am4c10507_si_001.pdf [file am4c10507_si_001.pdf]

Supporting Information for "Disentangling the Optoelectronic Behaviour of Lead Iodide Governed by Two-Dimensional Electron Confinement"

Hamida Gouadria,[†] Fernando Aguilar-Galindo,[‡,¶] Jesús Álvarez-Alonso,[†,§] Juan José de Miguel,[†,§] Sergio Díaz-Tendero,[*,‡,¶,§] and María José Capitán[*,‖]

†*Departamento de Física de la Materia Condensada, Universidad Autónoma de Madrid, 28049 Madrid, Spain.*

‡*Departamento de Química, Universidad Autónoma de Madrid, 28049 Madrid, Spain.*

¶*Institute for Advanced Research in Chemistry (IAdChem), Universidad Autónoma de Madrid, 28049 Madrid, Spain.*

§*Condensed Matter Physics Center (IFIMAC), Universidad Autónoma de Madrid, 28049 Madrid, Spain*

‖*Instituto de Estructura de la Materia IEM-CSIC, c/Serrano 121, 28006 Madrid, Spain.*

E-mail: sergio.diaztendero@uam.es; mj.capitan@csic.es

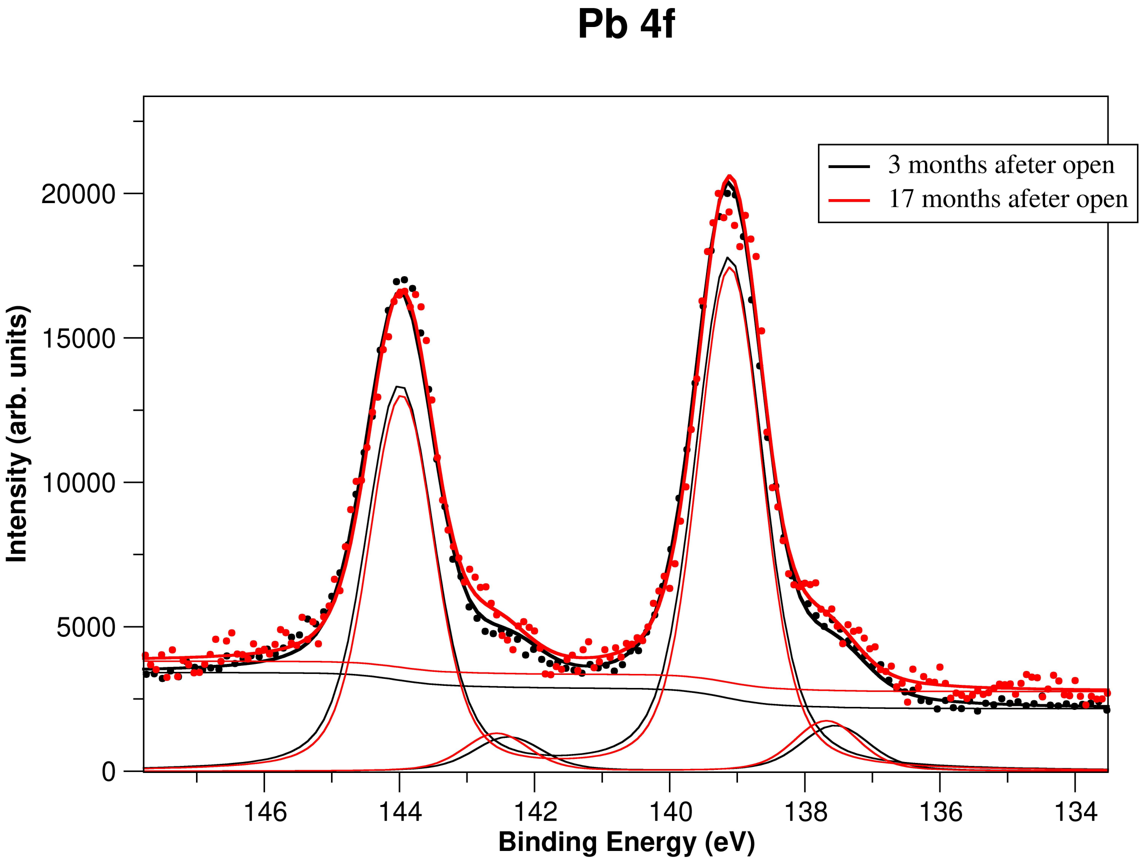

Figure S1: Pb 4f XPS data for the commercial PbI_2 sample at two different moments after the bottle opening. It can be observed that the spectra only depend on the time that the sample is set under vacuum and no on the time preserved the sample at atmospheric conditions. The experiments are measured within the first one after introducing under UHV environment.

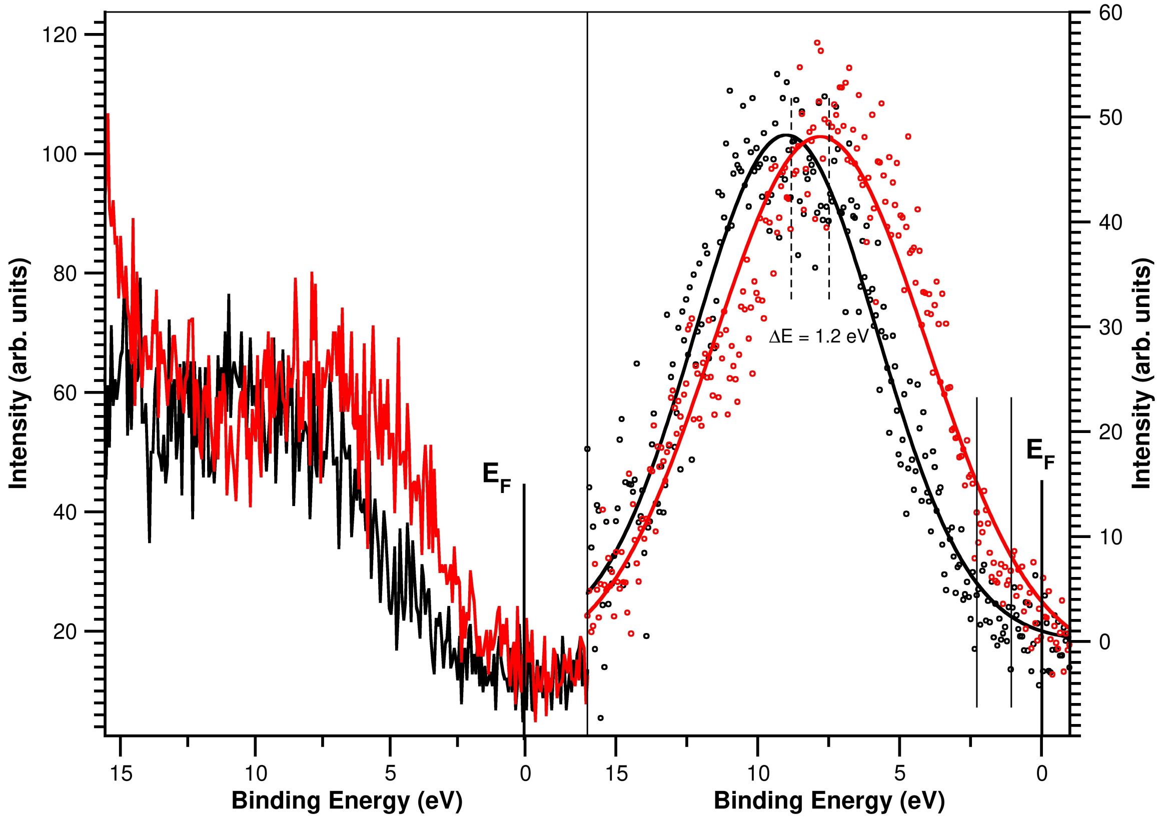

Figure S2: UPS measurement for fresh PbI_2 sample with/without visible light illumination (black/red lines respectively). On left, the raw data and on right data after exponential background subtraction and Gaussian fit.

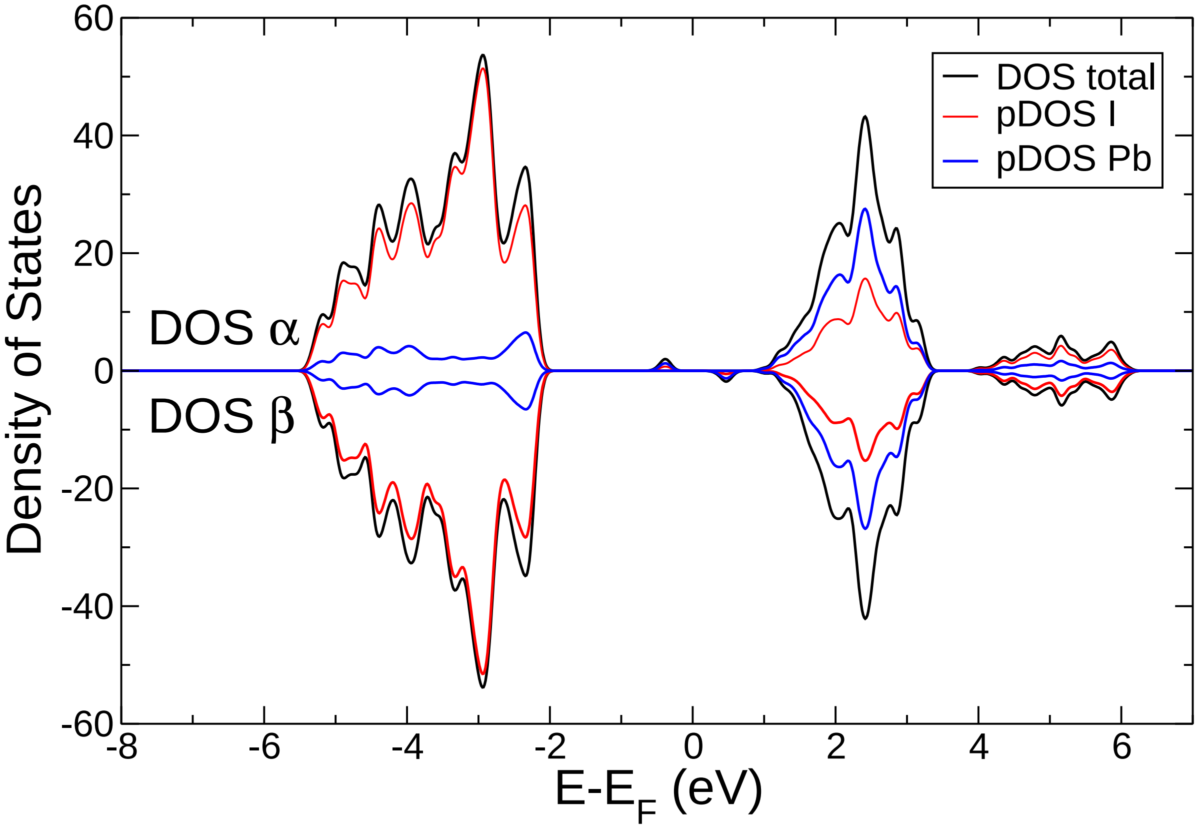

Figure S3: Density of states near the Fermi level, for the 3x3x3 bulk model including a vacancy, computed at the HSE level over the OPTPBE geometry. α and β electrons are represented with positive and negative signs respectively. Black line corresponds to the total DOS, red to the DOS projected on I atoms and blue to DOS projected on Pb atoms.

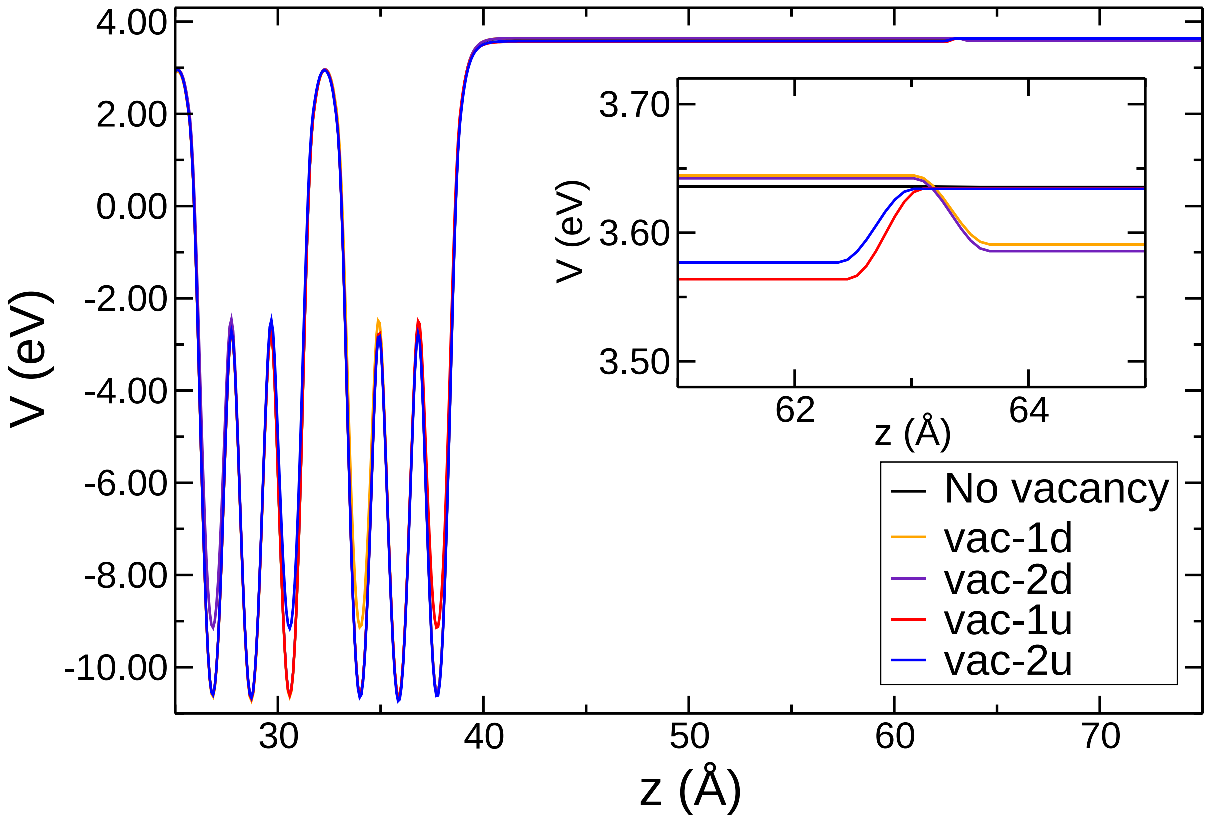

Figure S4: Averaged potential as a function of z computed at the OPTPBE level of theory. The inset shows the dipole correction. See Fig.10(a) in the main paper for the geometry of each model.

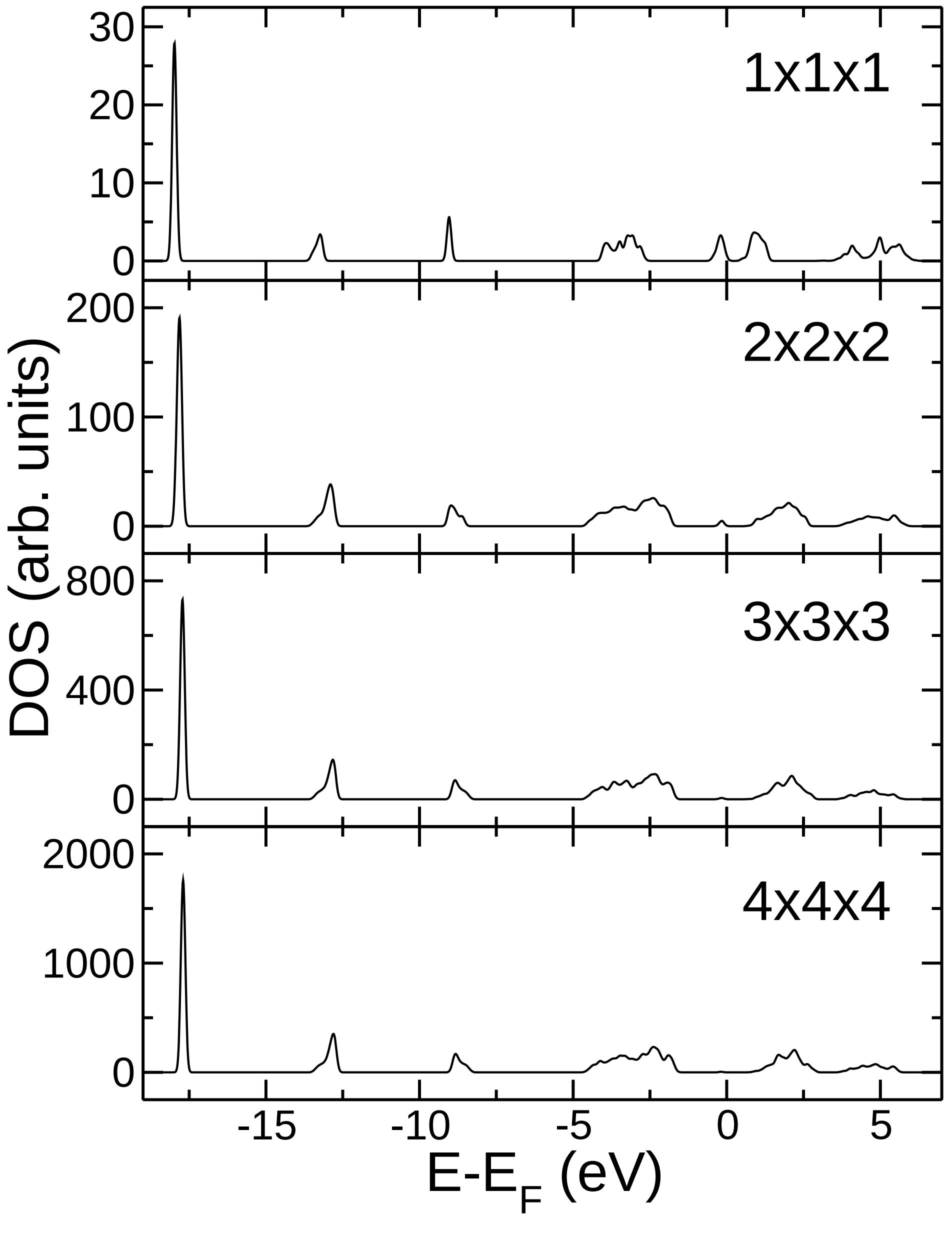

Figure S5: Density of states of bulk models with a vacancy for different cell sizes 1x1x1, 2x2x2, 3x3x3 and 4x4x4. In all cases one vacancy per unit cell. Computed with the OPTPBE functional.
